# STUDY DESIGN, PROTOCOL, AND HUMAN HEALTH EFFECTS OF PFAS IN THE TSUKUBA CITY COHORT

**DOI:** 10.1093/geroni/igae098.4082

**Published:** 2024-12-31

**Authors:** Heesoo Eun, Jieun Yoon, Mayumi Kameyama, Hiroshi Hata, Tetsuaki Arai, Tomohiro Okura, Hiroyuki Nishiyama

**Affiliations:** National Agriculture and Food Research Organization, Tsukuba, Ibaraki, Japan; Uiversity of Tsukuba, Tsukuba, Ibaraki, Japan; National Agriculture and Food Research Organization, Tsukuba, Ibaraki, Japan; National Agriculture and Food Research Organization, Tsukuba, Ibaraki, Japan; Institute of Medicine/University of Tsukuba, Tsukuba, Ibaraki, Japan; Institute of Medicine/University of Tsukuba, Tsukuba, Ibaraki, Japan; Institute of Medicine/University of Tsukuba, Tsukuba, Ibaraki, Japan

## Abstract

PFAS (perfluoroalkyl and polyfluoroalkyl substances) are toxic chemicals that are urgently regulated worldwide. While several adverse effects on the human body are known, including impacts on liver, kidney, and reproductive functions, the effects of 30 different multicomponent PFAS on the human body remain largely unexplored. Recently, it has been suggested that PFAS may affect Alzheimer’s disease, necessitating more rapid investigation. Given that PFAS exposure in humans primarily occurs through food intake, we initiated a cohort study in collaboration with various medical specialists to understand and address the actual situation of PFAS exposure in Japanese individuals who consume seafood with relatively high PFAS concentrations. The ongoing Tsukuba Happiness Life Study involves a large group of citizens aged 45 to 89 years from Tsukuba City, Ibaraki Prefecture. The study will investigate the molecular species and concentrations of more than 30 PFAS in blood and urine samples collected from 1,000 healthy, consenting participants. Additionally, cognitive function and motor performance will be assessed. Specialists from various medical departments at the University of Tsukuba Hospital will evaluate the results, relating the molecular species and concentrations of PFAS to their health effects. This research will, for the first time, reveal the effects of PFAS derived from dietary habits on the human body, providing comprehensive knowledge on PFAS, including their impact on cognitive and motor functions.

